# Water Salinity Should Be Reduced for Irrigation to Minimize Its Risk of Increased Soil N_2_O Emissions

**DOI:** 10.3390/ijerph15102114

**Published:** 2018-09-26

**Authors:** Qi Wei, Junzeng Xu, Linxian Liao, Yawei Li, Haiyu Wang, Shah Fahad Rahim

**Affiliations:** 1 State Key Laboratory of Hydrology-Water Resources and Hydraulic Engineering, Hohai University, Nanjing 210098, China; weiqi8855116@163.com; 2 College of Water Conservancy and Hydropower Engineering, Hohai University, Nanjing 210098, China; 3 College of Agricultural Engineering, Hohai University, Nanjing 210098, China; liaolinxian@hhu.edu.cn (L.L.); yaweizx@163.com (Y.L.); haiyu_wang@outlook.com (H.W.); fahadrahim@hotmail.com (S.F.R.)

**Keywords:** brackish water irrigation, N fertilizer, nitrogen oxide, water-filled pore space, electrical conductivity

## Abstract

To reveal the effect of irrigation salinity on soil nitrous oxide (N_2_O) emission, pot experiments were designed with three irrigation salinity levels (NaCl and CaCl_2_ of 1, 2.5 and 4 g/L equivalence, Ec = 3.6, 8.1 and 12.7 ds/m), either for 0 kg N/ha (N0) or 120 kg N/ha (N120) nitrogen inputs. N_2_O emissions from soils irrigated at different salinity levels varied in a similar pattern which was triggered by soil moisture dynamics. Yet, the magnitudes of pulse N_2_O fluxes were significantly varied, with the peak flux at 5 g/L irrigation salinity level being much higher than at 2 and 8 g/L. Compared to fresh water irrigated soils, cumulative N_2_O fluxes were reduced by 22.7% and 39.6% (N0), 29.1% and 39.2% (N120) for soils irrigated with 2 and 8 g/L saline water, while they were increased by 87.7% (N0) and 58.3% (N120) for soils irrigated with 5 g/L saline water. These results suggested that the effect degree of salinity on consumption and production of N_2_O might vary among irrigation salinity ranges. As such, desalinating brackish water to a low salinity level (such as 2 g/L) before it is used for irrigation might be helpful for solving water resources crises and mitigating soil N_2_O emissions.

## 1. Introduction

Due to a shortage of irrigation water, brackish water (salinity concentration of 2–5 g/L), or marginal saline water, has been adopted as an alternative of fresh water for agricultural irrigation in some areas of the world [1,2,3,4,5]. Prolonged application of brackish or saline water could exacerbate soil salinity, which inevitably affects soil properties, microbial activity, and thereby N transformation [6,7,8,9,10]. Meanwhile, agricultural soil is the main source of nitrous oxide (N_2_O), contributing to 59.4% of global anthropogenic source of N_2_O. The rise of atmospheric N_2_O concentrations is primarily due to unreasonable agricultural management activities [11,12]. Soil moisture affected by irrigation management has been demonstrated to be one of the most important factors associated with soil N_2_O emissions [13,14,15]. Therefore, it is expected that the interaction of soil salt and water will result in a different N_2_O emissions from soil [16].

Increasing salt-affected agricultural land has attracted interest to understand the influence of salinity on soil N_2_O emissions [17,18,19,20,21,22], but their conclusions sometimes conflict with each other. For example, Zhang et al. [22] found that N_2_O fluxes from a drip-irrigated cotton field without nitrogen (N) fertilizer application increased with irrigation salinity from 0.4 to 8.0 ds/m. Yet, other studies [16,20] showed that salinity reduced N_2_O emission significantly, either from soils or industrial waste-water sludge. Furthermore, some researchers [19,22] found that the relationship between salinity and N_2_O emissions varied among soil conditions. For instance, at under 65% water hold capacity (WHC), soil N_2_O fluxes at 11.7 g/L salinity level were greater than at 35.1 g/L, while it showed the opposite behavior at under 40% WHC [19]. Moreover, under 0 kg N/ha, N_2_O flux at 8.0 ds/m irrigation salinity level was greater than at 0.4 ds/m, but it showed opposite results under 360 kg N/ha [22]. In most of the literature regarding the impact of soil salinity on N_2_O emissions, few researchers have focused on N_2_O emissions from soils irrigated with brackish or saline water. The connection between N_2_O emissions and irrigation salinity is still unclear.

Thus, pot experiments were conducted on a silty clay soil treated with or without N application, under three irrigation salinity levels. The primary objective of this study was to quantify N_2_O fluxes from soils irrigated with water at different salinity levels, and discuss its responses to variations of soil moisture and salinity.

## 2. Experiments

### 2.1. Experimental Design

Experiments were conducted from May to August 2016 on a silty clay soil collected from a plastic greenhouse located at Nanjing Vegetables Scientific Institute in 2012 (31°56′ N, 118°37′ E). A soil sample was air-dried, ground, sieved, homogenized, and finally packed into cylindrical soil columns (I.D. = 30 cm, depth = 50 cm) to a bulk density of 1.25 g/cm^3^ for 0–5 cm layer and 1.33 g/cm^3^ for 5–50 cm layer, according to the local soil’s native bulk density. Columns were left undisturbed for over 60 days to avoid the influences of soil disturbance on N_2_O emission. Detailed soil properties are listed in Table 1.

The soil sample was irrigated at three salinity levels: 2, 5 and 8 g/L (namely treatments S2, S5 and S8, with Ec of 3.6, 8.1 and 12.7 ds/m), either for zero (N0) or field N application levels (120 kg N/ha, N120). Saline water was produced by adding NaCl and CaCl_2_ at a mass ratio of 1:1 to fresh water. Urea (N = 46.2%) was used as the N source. Fresh water (Ec = 0.3 ds/m), either with N0 and N120 application, was set as a control (namely treatments CK and CK + N). All treatments (S2, S5, S8, CK, S2 + N, S5 + N, S8 + N and CK + N) were replicated six times (three for gas sampling and another three for soil sampling). The soil was watered twice, at 22:00 on 19 July and 22:00 on 3 August 2016. For each treatment, 3530 and 3210 mL of fresh or saline water was supplied to replenish surface (0–20 cm) soil moisture to field capacity for the first and second irrigation. Air temperatures after the first irrigation ranged from 33.1 to 38.4 °C, which was on average 8.3% higher than that after the second irrigation (29.9–38.4 °C).

### 2.2. Gases Sampling and Analyzing

**Figure 1:**
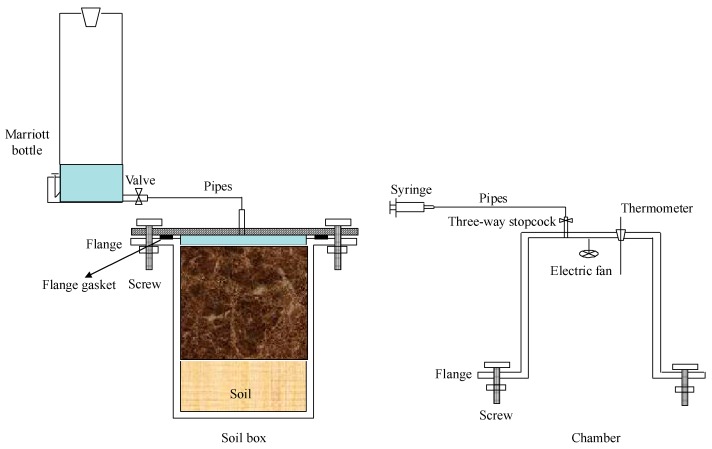
Sketch of soil box and the chamber.

Gas samples from each soil column were collected daily during the observation period using the static chamber (I.D. = 30 cm, depth = 30 cm), which was made of 8 mm polyvinyl chloride (PVC) and equipped with a thermometer and an electric fan at the top for measuring air temperature and mixing air inside (Figure 1). A rubber tube was inserted into the chamber from the top and connected to a three-way stopcock, which was used to draw gas samples using a 10 mL syringe. Four gas samples from each chamber were collected at 10-min intervals between 9:45 h and 10:15 h on each sampling day. When gas samples were collected, air temperatures inside the chambers, as well as soil temperature at 0, 5, 10 and 15 cm layers, were measured using mercury thermometers (accuracy, 0.1 °C).

N_2_O concentration was analyzed within 48 h after sampling using a gas chromatograph system (Agilent 7890B; Agilent Technologies Inc., Santa Clara, CA, USA) with an electron capture detector (ECD). N_2_O fluxes were calculated according to the linear increment in N_2_O concentration within the chamber [11]. Cumulative N_2_O fluxes were calculated by integrating daily values along the sampling period. 

### 2.3. Soil Moisture and Salinity Measurement

Soil samples (approximately 3–5 g each) from 20 cm depth in 5 cm increment were collected at 10:00 h every 2 days using stainless steel samplers (O.D. = 10 cm). Soil moisture content was determined gravimetrically by drying at 105 °C for 24 h, and water-filled pore space (WFPS) was calculated as the percentage of soil volumetric moisture content relative to the soil porosity. Soil electrical conductivity (Ec) was measured (soil:water = 1:5) using an electrical conductivity meter (TES-1381K, Taiwan, China). 

### 2.4. Statistical Analysis

Statistical analysis was performed using SPSS 19.0 (SPSS Inc., Chicago, IL, USA). A Fisher’s least significant difference (LSD) test was performed to determine the difference in soil average or cumulative N_2_O fluxes (*n* = 3) among treatments in non-pulse or pulse period. A paired *t* test was conducted to evaluate the difference among soil depths or treatments soil-related factors (temperature, moisture and salinity) with time series (*n* = *N*, the *N* was the number of data in each time series).

## 3. Results

### 3.1. Soil Temperature

**Figure 2:**
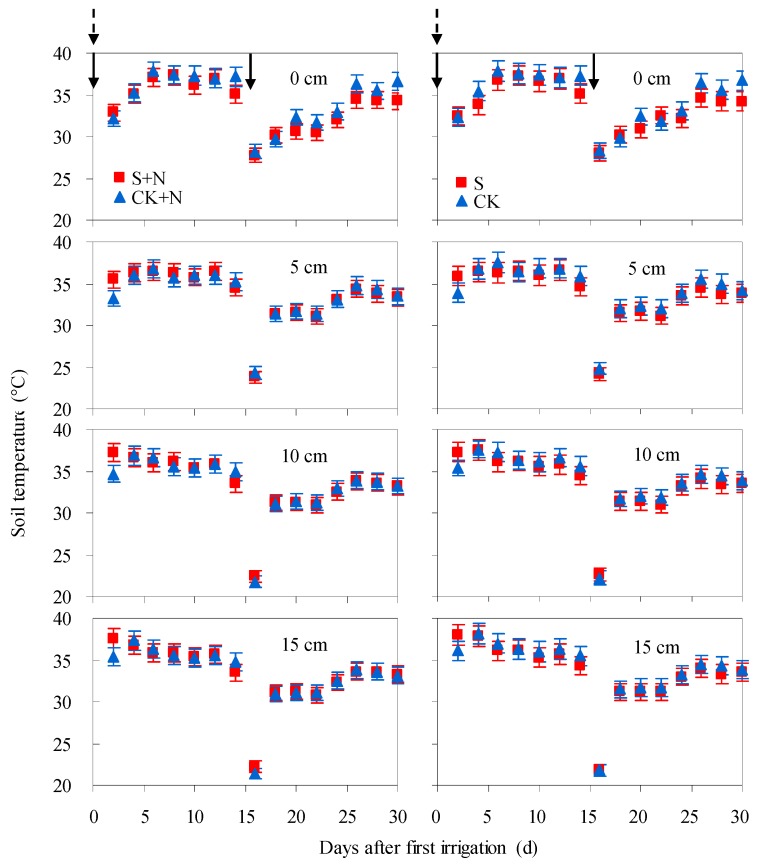
Soil temperature at 0, 5, 10 and 15 cm layers after two irrigation events from 20 July to 18 August. Solid arrow and dotted arrow represent irrigation and fertilization event, respectively. S + N and S means the average soil temperature from three saline water irrigated soils (S2, S5 and S8) with N120 and N0 application, respectively.

No significant differences were found in soil temperature among treatments with fresh or saline water irrigation (Figure 2). For all treatments, soil temperatures at 0, 5, 10, and 15 cm layers mainly fell within the ranges of 27.3–37.9 °C, 23.1–37.1 °C, 21.8–37.6 °C, and 21.4–38.0 °C, respectively. The minimum values were observed 2 days after the second irrigation due to the reduced air temperature and low irrigation water temperature (25.7 °C). Among soil depths, soil temperatures decreased with the increasing of soil layers, but the differences were mostly not significant (*p* > 0.1).

### 3.2. Soil Moisture

**Figure 3:**
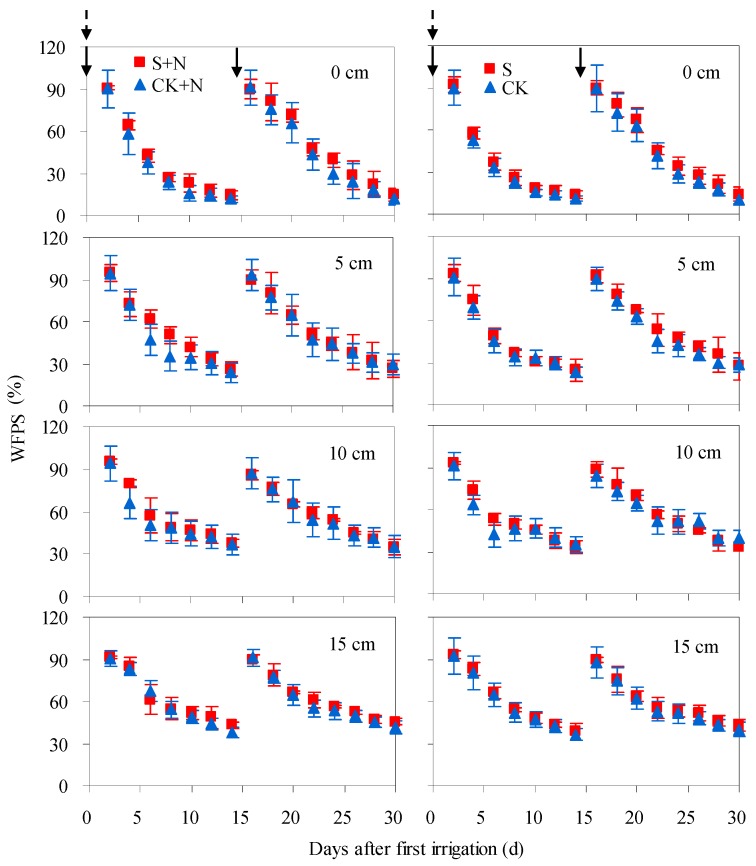
Soil WFPS at 0, 5, 10 and 15 cm depths after two irrigation events from 2 July to 18 August. Solid arrow and dotted arrow represent irrigation and fertilization event, respectively. S + N and S denotes the average WFPS from three saline water irrigated soils (S2, S5 and S8) with N120 and N0 application, respectively.

Soil moisture at 0–15 cm layers varied in the similar pattern and fell within same ranges in both saline water and fresh water irrigated soils, either under N0 or N120 level (Figure 3). Soil moisture varied significantly at early observation period, and then decreased with time from 89.8–93.8%, 90.9–95.9%, 91.1–96.3% and 90.2–94.4% WFPS to 11.7–17.2%, 23.3–32.0%, 32.7–39.4% and 40.1–46.4% WFPS at 0, 5, 10 and 15 cm layers, respectively. Among soil layers, soil WFPS varied more significantly in 0 and 5 cm depths than in 10 and 15 cm depths.

### 3.3. Soil Salinity

**Figure 4:**
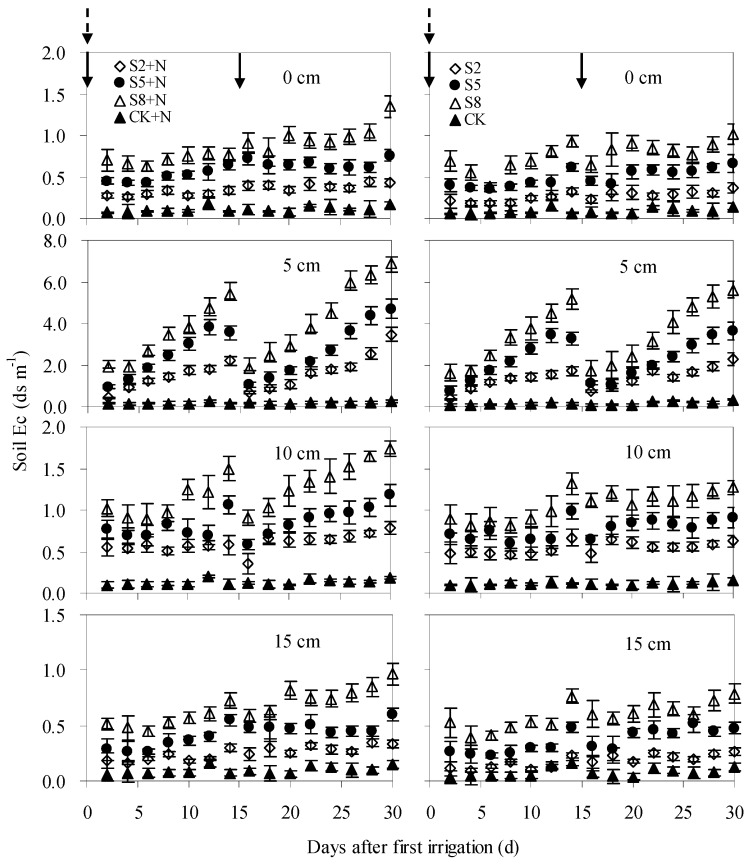
Soil Ec at 0, 5, 10 and 15 cm depths after two irrigation events from 2 July to 18 August. Solid arrow and dotted arrow represent irrigation and fertilization event, respectively. S2, S5 and S8 represent soils irrigated with water at 2, 5 and 8 g/L salinity level; S2 + N, S5 + N and S8 + N represent soils irrigated with water at 2, 5 and 8 g/L salinity level and nitrogen fertilization (120 kg N/ha) application. CK + N and CK represent soils irrigated with fresh water at N120 and N0 level, respectively.

In contrast to soil moisture, soil Ec was low immediately after irrigation and increased gradually along with the decrease of soil moisture (Figure 4). Generally, soil Ec at 5 cm depths was higher than at upper or lower depths, indicating that solute mostly accumulated in soil at a depth of 5 cm. Among soil layers, soil Ec from saline water irrigated soils was significantly higher (*p* < 0.05) than that of fresh water irrigated soils (0.06–0.2 ds/m), either under N0 or N120 level. For S2 + N, S5 + N and S8 + N treatments, soil Ec mainly varied in the ranges of 0.2–2.2 ds/m, 0.3–3.8 ds/m, 0.5–5.4 ds/m, respectively. It increased remarkably with irrigation salinity from 2 to 8 g/L, with averages from S5 + N and S8 + N treatments 59.1% and 139.8%, i.e., significantly higher than from S2 + N treatment (*p* < 0.05). For soils with N0 application (S2, S5 and S8 treatments), soil Ec was slightly lower than that of soils with N120 application, but varied in a similar pattern.

### 3.4. N_2_O Emission Fluxes

**Figure 5:**
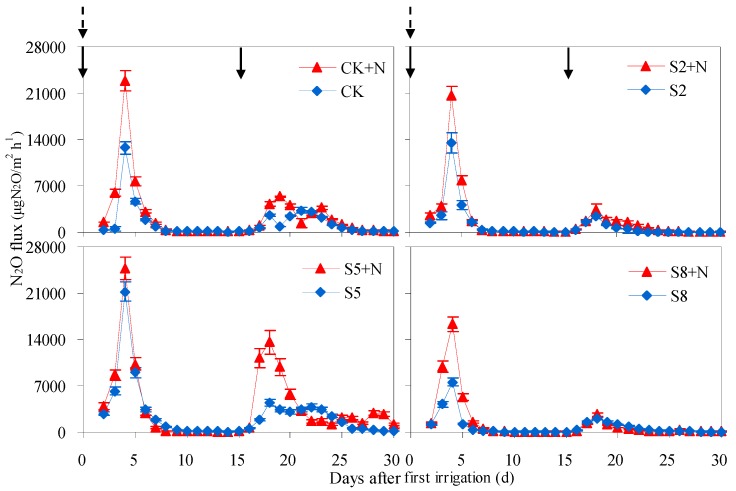
N_2_O emissions from soil irrigated at different salinity levels after two irrigation events from 20 July to 18 August. Solid arrow and dotted arrow represent irrigation and fertilization event, respectively. S2, S5, and S8 represent soils irrigated with water at 2, 5 and 8 g/L salinity level; S2 + N, S5 + N and S8 + N represent soils irrigated with water at 2, 5, and 8 g/L salinity level and nitrogen fertilization (120 kg N/ha) application. CK + N and CK represent soils irrigated with fresh water at N120 and N0 level, respectively.

Average or pulse N_2_O fluxes from saline water irrigated soils were quite different among salinity levels, even though their temporal courses triggered by the soil wetting-drying process were much similar. Under the N120 level, N_2_O fluxes from soil irrigated at 5 g/L salinity level were much higher than at 2 and 8 g/L levels after both irrigations. Under the N0 level, a similar phenomenon was found for soil N_2_O fluxes after first irrigation, yet it remained almost the same among salinity levels after the second irrigation (Figure 5). 

For S2 + N, S5 + N and S8 + N treatments, N_2_O fluxes increased rapidly during the first 3 days after first irrigation to 7812.4, 10,275.1, and 5353.2 μg N_2_O/(m^2^h), and then declined to 66.5, 41.9, and 71.7 μg N_2_O/(m^2^h). Irrigation significantly stimulated N_2_O emission from soils, with peak N_2_O fluxes after second irrigation 32.3–140.9 times greater than observed on 2 August prior to the second irrigation. Compared to CK + N treatment, the peak N_2_O flux was enhanced by 8.2% for S5 + N treatment, while it was reduced by 10.2% and 28.8% for S2 + N and S8 + N treatments. As a result, the average N_2_O flux from S5 + N treatment increased by 1406.1 μg N_2_O/(m^2^ h), and from S2 + N and S8 + N treatments decreased by 702.1 and 944.7 μg N_2_O/(m^2^h), compared to CK + N (Table 2). 

For S2, S5 and S8 treatments, pulse N_2_O fluxes were observed 3 days after both irrigations, primarily in the ranges of 7590.6–21,245.7 μg N_2_O/(m^2^h) and 2078.2–4424.2 μg N_2_O/(m^2^h). The peak N_2_O fluxes from S2 and S5 soils were 5.7% and 66.5% higher than CK, yet the flux from S8 soil was 40.5% lower. Consequently, the average N_2_O fluxes from S2 and S8 treatments were reduced by 22.6% and 39.6%, while from S5 treatment was increased by 87.7% (*p* < 0.01), compared to CK. Across salinity levels, N_2_O fluxes were significantly lower in unfertilized soils, decreasing by 39.1% on average (*p* < 0.05) (Table 2).

### 3.5. Cumulative N_2_O Emissions

**Figure 6:**
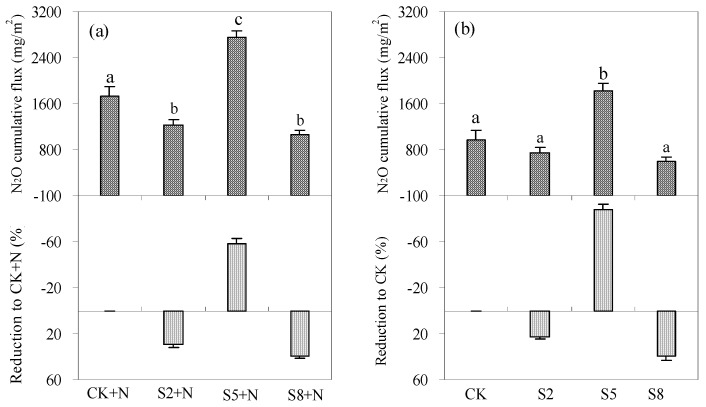
Cumulative N_2_O fluxes from saline water irrigated soils and its reduction rates compared to fresh water irrigated soils. (**a**) represent cumulative N_2_O fluxes from S2 + N, S5 + N, and S8 + N soils and its reduction rates compared to CK + N soils. (**b**) represent cumulative N_2_O fluxes from S2, S5, and S8 and its reduction rates compared to CK soils. Different letters on each bar represent significant difference in N_2_O emissions at *p* < 0.05 level. S2, S5, and S8 represent soils irrigated with water at 2, 5, and 8 g/L salinity level; S2 + N, S5 + N, and S8 + N represent soils irrigated with water at 2, 5, and 8 g/L salinity level and nitrogen fertilization (120 kg N/ha) application. CK + N and CK represent fresh water irrigated soils with or without nitrogen fertilization (120 kg N/ha) application, respectively.

Over the whole experimental period (30 days), cumulative N_2_O fluxes from saline water irrigated soils under N0 level ranged from 586.3 to 1822.4 mg/m^2^, with fluxes at 5 g/L salinity level as the highest (Figure 6). The application of exogenous N enhanced N_2_O emissions remarkably; the cumulative N_2_O fluxes from saline water irrigated soils under N120 level were 50.8–80.2% greater than under N0 level. Furthermore, under both N levels, cumulative N_2_O fluxes from 2 and 8 g/L saline water irrigated soils were significantly lower than from fresh water irrigated soils, decreasing on average by 25.9% and 39.4% (*p* < 0.05). Yet for 5 g/L saline water irrigated soil, it increased by 73.0% on average. 

## 4. Discussion

Both natural rainfall and irrigation can be important regulators of soil N_2_O emissions [23,24,25,26,27,28], causing large pulses emissions relative to normal conditions [29,30]. The occurrences of short-term pulse N_2_O emissions during drying processes were confirmed from saline water irrigated soils in the current experiment (Figure 5). The peak times, as well as the magnitudes of pulse N_2_O fluxes, were in agreement with the results reported in the aforementioned literature. Moreover, correlation analysis between N_2_O emissions and soil moisture or salinity indicated that N_2_O fluxes from different saline water irrigated soils increased exponentially with the increase of soil WFPS (Table 3). The determined coefficients of the exponential functions between N_2_O flux and soil WFPS in saline water irrigated treatments were higher than fresh water irrigated treatments, with the determined coefficient for the treatment at low irrigation salinity level as the highest. Yet, when it comes to the factor of soil Ec, N_2_O fluxes was found to decrease exponentially with the increase of soil Ec in all treatments (Table 3). The determined coefficients decreased with irrigation salinity from 2 to 8 g/L. Generally, the determined coefficients of N_2_O flux to soil WFPS were much higher than it to soil Ec. These results suggested the dynamic of N_2_O emissions from soils irrigated with saline water were significantly influenced by both soil WFPS and soil Ec.

Generally, pulse N_2_O emissions appeared when soil moisture, salinity and temperature were appropriate (Table 4). In current research, the pulse N_2_O fluxes under both N application levels (N0 and N120) were much higher from soils irrigated at 5 g/L salinity level than at 2 and 8 g/L, while conditions of soil WFPS and temperature kept almost the same ranges (Table 4). It implied that irrigation salinity was a key factor that affected the magnitudes of pulse N_2_O fluxes from saline water irrigated soils, and the exogenous N almost did not change the response of N_2_O peak fluxes to soil-related factors (soil moisture, Ec or temperatures).

Previous researches had investigated the impact of salinity on N_2_O emissions, whereas their conclusions were contradictory to each other [17,19,21,22,31] (Table 5). Sometimes it increased remarkably with the increase of salinity level [22], but sometimes reduced [17], or showed no effect [19,21,31]. In present study, N_2_O fluxes from 2 and 8 g/L (Ec of 3.6 and 12.7 ds/m) saline water irrigated soils were significantly lower than from fresh water irrigated soils (Ec of 0.3 ds/m), while from 5 g/L (Ec of 8.1 ds/m) saline water irrigated soil, it enhanced remarkably. This phenomenon likely suggested that the effect degree of salinity on consumption and production of N_2_O might vary among irrigation salinity ranges. Some previous results once indicated that the activity of N_2_O reductase, which reduces N_2_O to N_2_ in nitrogen transformation, was inhibited to a lesser degree under low rather than medium salinity [8,9,21,22,32,33,34]. That partially accounted for the higher N_2_O emission at moderate rather than low salinity levels. Furthermore, the activities of ammonia-oxidizing bacteria and ammonia-oxidizing archaea were significantly reduced under high salinity levels, which may result in the inhibition of the first step of the nitrification (ammonia oxidation) process, and thus, possible reduced N_2_O emission levels [35]. This might be another important reason for decreased N_2_O emissions under high salinity level. These results also indicated that reducing salinity of brackish water from 5 to 2 g/L could be helpful for N_2_O mitigation.

With increasing shortage of agricultural water resources, brackish water (salinity concentration of 2–5 g/L) is widely applied for irrigation [2,22,36]. The current research was performed to investigate N_2_O emissions from soils irrigated with water at different salinity levels. Results indicated that the response of N_2_O emissions to salinity might vary among irrigation salinity ranges, and irrigation with desalinated brackish water could be a potential measure for N_2_O mitigation. But unfortunately, the N_2_O emissions were collected from a soil with low initial salinity (Ec = 0.4 ds/m), and the irrigation salinity level did not set in a finer classification (such as 2.5, 3, 3.5 and 4 g/L). Thereafter, more investigation should be done to reveal the effect of salts on N_2_O emissions under different initial soil salinity conditions and higher irrigation salinity levels, and to figure out a suitable irrigation salinity level, which is helpful for mitigating N_2_O emissions while meeting the requirement of crop growth.

## 5. Conclusions

Pulse N_2_O emissions from soils irrigated with saline water were found to be triggered by soil moisture dynamics, and the magnitude of pulse N_2_O fluxes for 5 g/L treatment were significantly higher than those for 2 and 8 g/L treatments. These results implied that soil wetting-drying processes dominated the temporal process of N_2_O emissions from saline water irrigated soils, while irrigation salinity and N level affected its magnitudes remarkably. Compared to fresh water irrigation, 2 and 8 g/L saline water resulted in lower N_2_O emissions from soils either under N0 or N120 level, while 5 g/L saline water irrigation enhanced it significantly. It can be inferred that the sensitivity of N_2_O production and consumption to irrigation salinity might be different among salinity ranges, and reducing the salinity of brackish water might be a potential measure for solving agricultural water crises and reducing soil N_2_O emissions.

## Figures and Tables

**Table 1:** Soil physical and chemical properties (mean ± standard deviation).

Characteristic	Value
Clay (%)	29 ± 1.1
Silt (%)	48 ± 1.7
Sand (%)	23 ± 0.9
Ec (ds/m)	0.4 ± 0.06
pH (H_2_O)	6.2 ± 0.1
Organic matter (OM, g/kg)	25.2 ± 0.3
Total nitrogen (TN, g/kg)	1.4 ± 0.02
Total phosphorus (TP, g/kg)	0.04 ± 0.01
Nitrate nitrogen (NO_3_^−^-N, mg/kg)	34.2 ± 2.9
Ammonium nitrogen (NH_4_^+^-N, mg/kg)	8.8 ± 0.7

**Table 2:** Average N_2_O fluxes from different treatments during pulse and non-pulse periods.

Treatments (*n* = 3)	N_2_O Fluxes (μg N_2_O/(m^2^h))				
	Pulse Period	Non-Pulse Period	Pulse Period	Non-Pulse Period	The Whole Period
	20 July–26 July	26 July–2 August	2 August–9 August	9 August–18 August	20 July–18 August
S2 + N	5305.9 ± 432.5a	100.0 ± 21.2a	1687.9 ± 252.9a	181.8 ± 22.7a	1709.8 ± 142.4a
	(72.4%)	(1.4%)	(23.0%)	(3.2%)	(100%)
S5 + N	7346.6 ± 637.4b	121.0 ± 27.8a	6577.8 ± 656.3b	1802.4 ± 203.5b	3818.0 ± 324.8b
	(44.9%)	(0.7%)	(40.2%)	(14.2%)	(100%)
S8 + N	5024.2 ± 341.7a	79.9 ± 16.4a	958.6 ± 90.4c	175.0 ± 21.0a	1467.2 ± 101.7a
	(79.9%)	(1.3%)	(15.2%)	(3.6%)	(100%)
CK + N	6132.1 ± 475.8a	181.1 ± 23.1b	2770.2 ± 213.7d	974.6 ± 67.4c	2411.8 ± 159.9c
	(60.4%)	(1.9%)	(32.5%)	(5.2%)	(100%)
S2	3353.1 ± 245.9a	77.5 ± 11.2a	969.8 ± 78.4a	54.9 ± 11.2a	1043.2 ± 95.8a
	(75.0%)	(1.7%)	(21.7%)	(1.6%)	(100%)
S5	6476.8 ± 432.1b	136.2 ± 21.0ba	2938.4 ± 202.6b	1008.1 ± 104.3b	2531.1 ± 286.7b
	(59.7%)	(1.2%)	(27.1%)	(12.0%)	(100%)
S8	2108.6 ± 157.9a	64.8 ± 11.8a	1134.2 ± 107.0a	141.6 ± 20.6a	814.3 ± 78.5a
	(60.4%)	(1.9%)	(32.5%)	(5.2%)	(100%)
CK	3042.3 ± 272.3c	110.4 ± 14.0b	1863.3 ± 126.2c	594.3 ± 32.4c	1348.7 ± 91.4a
	(52.6%)	(1.9%)	(32.3%)	(13.2%)	(100%)

Different letters in each column indicate significant differences at the 5% level. Values in the bracket represent the contribution to the total N_2_O emissions. S2, S5, and S8 represent soils irrigated with water at 2, 5 and 8 g/L salinity level; S2 + N, S5 + N and S8 + N represent soils irrigated with water at 2, 5 and 8 g/L salinity level and nitrogen fertilization (120 kg N/ha) application.

**Table 3:** The relationship of N_2_O emissions to soil moisture or salinity for each treatment.

Treatments	N_2_O V.S. WFPS		N_2_O V.S. Ec	
	Relation	Determine Coefficient (R^2^)	Relation	Determine Coefficient (R^2^)
CK + N	y = 13.21e^0.04x^	0.28 *	y = 2474e^−11.70x^	0.07 ^ns^
S2 + N	y = 12.50e^0.07x^	0.66 *	y = 14966e^−5.84x^	0.59 *
S5 + N	y = 76.87e^0.05x^	0.34 *	y = 41004e^−3.43x^	0.56 *
S8 + N	y = 20.41e^0.05x^	0.47 *	y = 6988e^−1.94x^	0.45 *
CK	y = 87.46e^0.04x^	0.26 *	y = 2980e^−15.53x^	0.15 ^ns^
S2	y = 10.82e^0.06x^	0.58 *	y = 42015e^−8.69x^	0.57 *
S5	y = 60.36e^0.05x^	0.44 *	y = 56362e^−4.26x^	0.55 *
S8	y = 14.75e^0.05x^	0.57 *	y = 14766e^−2.73x^	0.61 *

The symbols of * indicate correlation is significant at *p* < 0.05 level. ^ns^ represents no significance. S2 + N, S5 + N and S8 + N represent soils irrigated with water at 2, 5, and 8 g/L level and nitrogen fertilization (120 kg N/ha) application. S2, S5, and S8 represent soils irrigated with water at 2, 5, and 8 g/L salinity level. CK + N and CK represent treatments with fresh water irrigation under N120 and N0 level, respectively.

**Table 4:** Pulse N_2_O flux and its corresponding soil WFPS, Ec, and temperature.

Irrigation Events	Treatment	N_2_O Peak Fluxes (μg N_2_O/(m^2^h))	WFPS (%)	Ec (ds/m)	T (°C)
After first irrigation (20 July–1 August)	S2 + N	20,509.1 ± 1095.9	74.6 ± 6.1	0.47 ± 0.08	36.8 ± 0.4
	S5 + N	24,795.4 ± 1178.5	74.4 ± 5.9	0.68 ± 0.10	36.4 ± 0.5
	S8 + N	16,312.2 ± 808.4	76.3 ± 6.3	0.99 ± 0.12	36.7 ± 0.3
	CK + N	22,926.2 ± 1252.9	72.1 ± 5.6	0.10 ± 0.03	37.0 ± 0.4
	S2	13,492.5 ± 618.9	70.9 ± 5.7	0.41 ± 0.06	36.3 ± 0.3
	S5	21,245.7 ± 1139.9	71.5 ± 5.9	0.63 ± 0.09	36.4 ± 0.4
	S8	7590.6 ± 322.3	72.4 ± 5.3	0.87 ± 0.12	37.0 ± 0.4
	CK	12,759.9 ± 631.7	68.9 ± 6.3	0.07 ± 0.02	37.3 ± 0.6
After second irrigation (2 August–18 August)	S2 + N	3465.7 ± 589.0	75.2 ± 6.1	0.55 ± 0.08	31.0 ± 0.3
	S5 + N	13,619.1 ± 1359.9	77.8 ± 5.9	0.79 ± 0.11	30.9 ± 0.2
	S8 + N	2655.8 ± 251.0	78.9 ± 4.3	1.22 ± 0.10	31.1 ± 0.4
	CK + N	5418.9 ± 275.6	73.6 ± 6.0	0.10 ± 0.02	31.3 ± 0.3
	S2	2347.0 ± 223.2	76.8 ± 5.6	0.54 ± 0.08	31.1 ± 0.4
	S5	4424.2 ± 359.9	75.7 ± 6.3	0.64 ± 0.09	31.1 ± 0.3
	S8	2078.2 ± 275.6	78.4 ± 7.1	1.14 ± 0.12	31.4 ± 0.3
	CK	3304.2 ± 287.9	69.1 ± 6.8	0.07 ± 0.03	32.1 ± 0.5

The values of soil WFPS, Ec, and temperature represent the averages of those values at 0–15 cm layers. S2, S5, and S8 represent soils irrigated with water at 2, 5, and 8 g/L salinity level; S2 + N, S5 + N and S8 + N represent soils irrigated with water at 2, 5, and 8 g/L level and nitrogen fertilization (120 kg N/ha) application. CK + N and CK represent fresh water irrigated soils with or without nitrogen fertilization (120 kg N/ha) application, respectively.

**Table 5:** Summary of results of the effect of salinity on N_2_O emissions.

Experiment Type	Salt Type	Salinity Level	N Level (kg N/ha)	Cumulative N_2_O Flux	Conclusion	Reference
A pot experiment (30 d)	Nacl:Cacl_2_ (1:1)	2, 5 and 8 g/L in water (S2, S5 and S8)	0	5.9–18.2 kg/ha	S5 > CK > S2 > S8	Current research
			120	10.6–27.5 kg/ha	S5 + N > CK + N > S2 + N > S8 + N	
A incubation experiment (28 d)	NaCl + NH_4_Cl	0, 5.9, 11.7, 23.4 and 35.1 g/L in soil solution (S0, S5.9, S11.7, S23.4 and S35.1)	/	96.3–169.6 ug/(g soil)	65% WHC: S5.9 > S11.7 > S35.1 > S0 > S23.4 (ns)	[19]
	Na_2_SO_4_ + (NH_4_)_2_SO_4_		/	205.5–322.7 ug/(g soil)	65% WHC: S0 > S5.9 > S35.1 > S23.4 > S11.7 (ns)	
	Ca(NO_3_)_2_	0, 5.9, 17.6 and 29.3 g/L in soil solution (S0, S5.9, S17.6 and S29.3)	/	122.5–699.8 ug/(g soil)	70% WHC: S5.9 > S0 > S17.6 > S29.3 (ns)	
A incubation experiment	Nacl	2.5, 7.5, 10 g/L in water (S2.5, S7.5 and S10)	/	/	S10 (5.1) > S7.5 = S2.5 (2.2–2.3) (unit: mg/L)	[21]
A incubation experiment	Nacl	0, 1, 3 and 6 g/(kg soil) (S0, S1, S3 and S6)	/	/	20% moisture (*w*/*w*): S6 (0.5) < S3 (1.2) < S1 (2.8) (unit: ng/(kg h))	[17]
A bean field experiment (84 d)	Nacl	0.03 and 0.6 g/L in water (S0.03 and S0.6)	120	0.5 and 0.4 kg/ha	S0.03 > S0.6 (ns)	[31]
			25	0.3 and 0.4 kg/ha	S0.03 < S0.6 (ns)	
A cotton field experiment (57 d)	Nacl:Cacl_2_ (1:1)	0.4, 4.6 and 8.1 ds/m in water (C0.4, C4.6 and C8.1)	0	0.1–~0.2 kg/ha	C0.4 < C4.6 < C8.1	[22]
			360	0.3–0.4 kg/ha	C8.1 < C0.4 < C4.6	

ns means the difference in N_2_O fluxes between treatments was not significant.
